# *Operando* NMR Monitoring of Electrochemical Reactions

**DOI:** 10.1021/acs.oprd.5c00483

**Published:** 2026-04-23

**Authors:** Ken S. Lee, Federico Barbieri, Colin S. Crawford, Heike Hofstetter, Jennifer M. Schomaker

**Affiliations:** † Department of Chemistry, 5228 University of Wisconsin-Madison, 1101 University Avenue, Madison, Wisconsin 53706, United States; ‡ Department of Chemistry, 230170 University of Pavia, Pavia, Lombardy 12-27100, Italy

**Keywords:** NMR, *operando*, electrochemistry, electrochemical reactions, kinetics

## Abstract

Electrochemical methods enable the controlled generation of reactive radical intermediates, yet mechanistic interrogations of such transformations remain challenging due to the dynamic, heterogeneous nature of these systems. Here we report a simple, commercially accessible *operando* electrochemical NMR (EC-NMR) platform adapted from a Bruker InsightMR® flow unit for real-time monitoring of electrochemical reactions. Integration of a batch electrochemical cell with an NMR flow tube allows continuous circulation and quantitative acquisition of ^1^H NMR spectra under the reaction conditions. This analytical tool helped to elucidate the chemoselectivity of (2,2,6,6-tetramethylpiperidin-1-yl)­oxyl (TEMPO)-mediated allene dioxygenation and revealed off-cycle pathways in the Shono oxidation. Extended monitoring of an electroreductive imine coupling further demonstrated the robustness of this approach. Overall, *operando* EC-NMR offers a powerful tool for kinetic analysis in synthetic electrochemistry.

## Graphical abstract

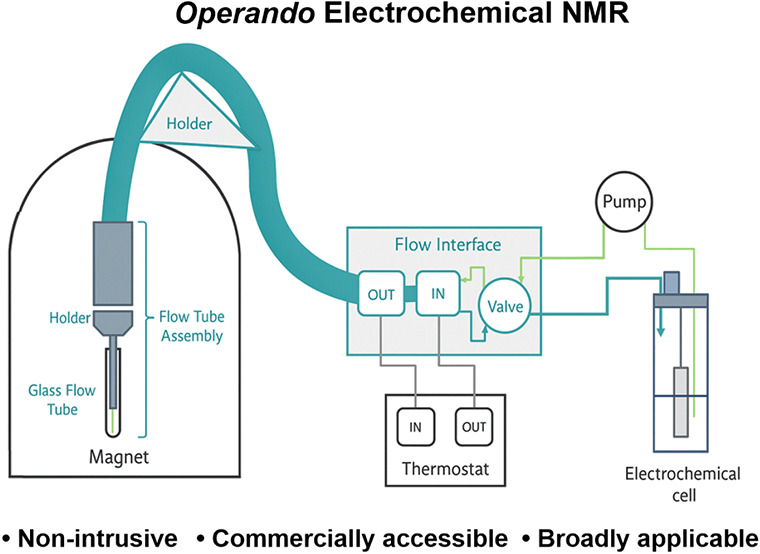

## Introduction

Electrochemistry has recently emerged as a powerful strategy in synthetic chemistry for the construction of valuable chemical motifs.^1^ The ability to generate highly reactive radical and radical ion intermediates in a controllable manner under mild, environmentally friendly conditions has inspired the development of numerous electrochemical methodologies for diverse transformations. The use of redox-active catalysts and electrocatalytic mediators further enables precise control over these reactive species, enhancing both selectivity and efficiency. A detailed mechanistic understanding of these systems would greatly accelerate the rational design and optimization of electrochemical reactions.^2^ Electroanalytical tools such as cyclic voltammetry (CV),^3^ pulsed voltammetric techniques, rotating disk electrodes, scanning electrochemical microscopy (SECM),^4^ and spectroelectrochemistry^5^ have provided a multitude of possibilities for investigating mechanisms in these complex systems.^6^ Moreover, machine learning algorithms can process data from CVs in a high-throughput manner to aid in the analysis of electrochemical systems with minimal human interference.^7^ However, because electrochemical reactions often proceed through various reaction pathways that give rise to short-lived radical intermediates, the development of real-time analytical techniques capable of probing these dynamic, heterogeneous systems remain critical for mechanistic studies.^8^

The term “*operando* spectroscopy” was initially coined by Bañares in 2002 and refers to an analytical approach where spectroscopic characterization occurs simultaneously with reaction progress.^9^ Since that time, the scientific community has developed *operando* techniques for NMR, IR, Raman, mass spectrometry (MS), UV–vis, and calorimetry as powerful tools for mechanistic investigation.^10^ Among these, NMR spectroscopy is particularly attractive due to its unparalleled chemical specificity that enables noninvasive, quantitative differentiation of individual nuclei, making it aptly suited for *operando* analysis.^11^ Specifically, electrochemical NMR (EC-NMR) has been employed to study electrocatalytic processes since the early 1990s and has been comprehensively reviewed by the Tong and Zhao groups.^12,13^ However, previous setups of *operando* EC-NMR required either the integration of specialized hardware or different nuclear polarization techniques to accommodate the heterogeneous nature of electrochemical systems. In 2019, the Ackermann group introduced the first continuous microflow *operando* EC-NMR platform to monitor a rhodium-electrocatalyzed alkyne annulation reaction.^14^ This system circumvented the need for extensive hardware modification by interfacing the NMR spectrometer with an electrochemical flow cell via a peristaltic pump, enabling nonintrusive collection of kinetic data. Thus, the further development of a simple, commercially available design for *operando* EC-NMR would significantly expand the usability of this powerful methodology across both academic and industrial research settings.

Our group recently developed an electrooxidative allene dioxygenation reaction that converts aryl allenes **1** into either the desired allylic alcohol **2** or a diene byproduct **3** (Scheme 1A).^15^ While a plausible mechanism for this transformation was proposed (Scheme 1B), we were unable to effectively monitor the reaction progress to obtain details of the competing formation of **2** and **3**.

**Scheme 1:**
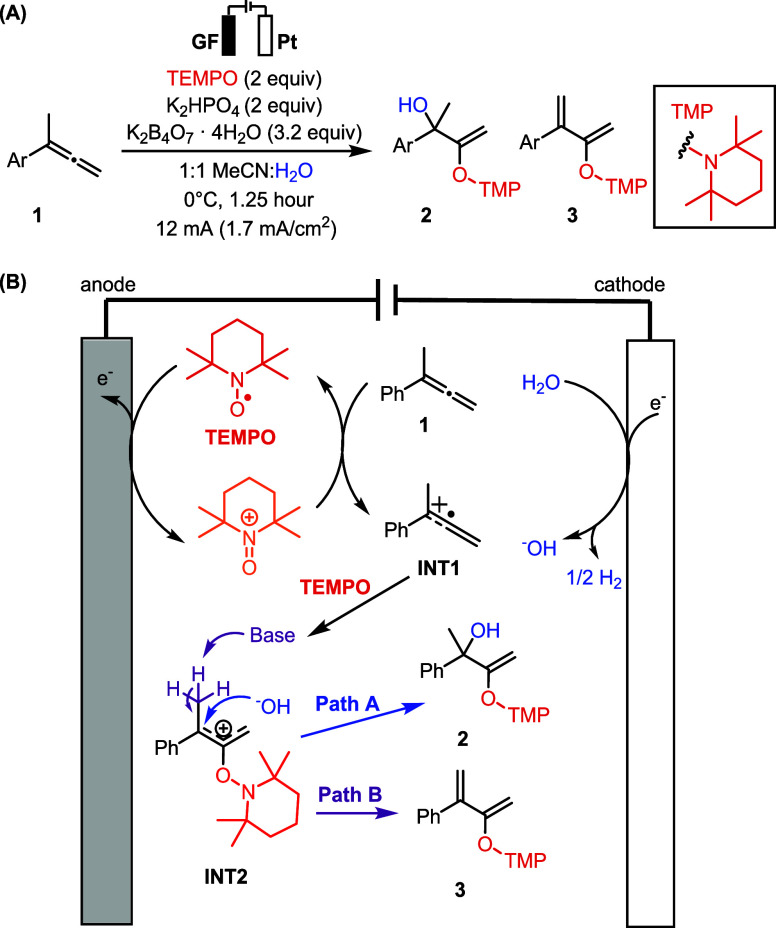
(A) TEMPO-Mediated Electrooxidative Dioxygenation of Aryl Allenes; (B) Proposed Mechanism of Allene Dioxygenation

To address our inability to effectively monitor these electrochemical reactions, we designed a simple *operando* EC-NMR experiment by repurposing a Bruker InsightMR® flow unit.^16^ Seamless integration of the batch electrochemical cell with an NMR flow tube that can be swapped out easily in a 500 MHz NMR furnished a nonintrusive *operando* EC-NMR system.^17^ This configuration enabled access to real-time kinetic data of selected electrochemical reaction systems using dedicated InsightMR® processing software.

## Results and Discussion

### Experimental Design

The InsightMR® flow unit component developed in this study enables temperature-controlled circulation of the reaction mixture between the electrochemical vessel and the NMR spectrometer for *operando* monitoring. A pump continuously transports the solution from the batch electrochemical cell through temperature-regulated tubing into an NMR flow tube (Figure 1), after which the solution is recirculated back to the reaction vessel. In this system, both the temperature and flow rate are readily adjustable. Real-time data are collected as 1-scan ^1^H NMR spectra using the InsightMR® software package, which also permits “on-the-fly” modifications to acquisition parameters and automatically updates queued experiments. Processed data can be visualized as stacked plots in MestReNova or another suitable analysis software.^18^ Electrochemical reactions were conducted in a Schlenk tube connected to a nitrogen balloon. The tube was sealed with a septum punctured by two electrode holders containing their respective electrodes. All NMR measurements were acquired on a Bruker 500 MHz spectrometer equipped with a Prodigy BroadBand Observe (Prodigy BBO) probe.

**Figure 1:**
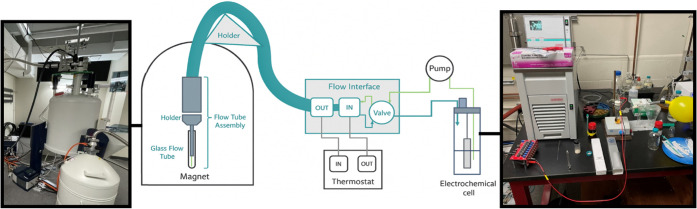
EC InsightMR® flow unit.

### Allene Dioxygenation

In our electrooxidative allene dioxygenation reaction, we proposed TEMPO-mediated oxidation of the allene **1** yields allylic cation **INT2** (Scheme 1B, *vide supra*).^15^ Subsequent hydroxylation or a competing deprotonation pathway furnishes products **2** and **3**, respectively. The *operando* EC-NMR system described above was used to probe product formation and chemoselectivity between these two reaction pathways. After adapting the reaction to a recirculating flow-electrochemistry^19^ setup, the experiment was carried out in 20:1 *d*_3_-MeCN:D_2_O, collecting ^1^H NMR spectra at 1 min intervals over the course of 1.5 hours to track the formation of **2** and **3** in real time.

The *operando* data revealed that the allylic alcohol **2** appeared within the first 5 min of electrolysis, while the diene **3** was not detected until approximately 7 min into the reaction (Figure 2A). A stacked plot of the recorded spectra showed the expected decrease in starting material **1**, concomitant with the gradual growth of both **2** and **3** over the course of the reaction (Figure 2B). These findingsagreed withthe bulk electrolysis results, which exhibited higher selectivity for **2** at lower temperatures (0 °C). Collectively, these findings demonstrate the value of *operando* EC-NMR for obtaining mechanistic insights and optimizing reaction conditions in real time. To further highlight the versatility of this analytical platform, two additional representative electrochemical transformations were also investigated.

**Figure 2:**
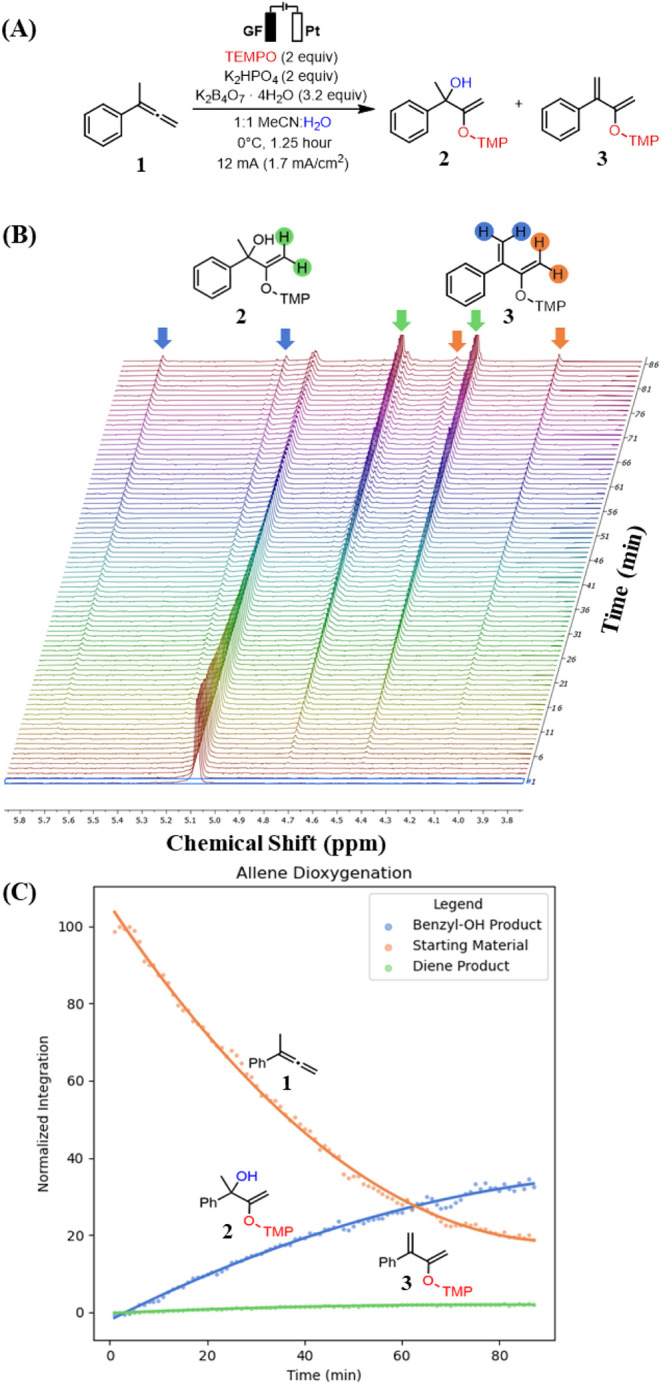
(A) TEMPO-mediated electrooxidative dioxygenation of **1**. (B) Stacked spectra of electrooxidative dioxygenation of **1**. (C) Normalized integral graph of stacked spectra.

### Shono Oxidation

In 1975, Shono and coworkers reported the anodic oxidation of cyclic and acyclic amides and carbamates in polar solvents to form *N,O*-acetals, a transformation now considered foundational in organic electrochemistry.^20^ Since then, the Shono oxidation has been extensively optimized to enable highly regio- and stereocontrolled oxidation processes.^21^ The resulting *N*,*O*-acetals are versatile intermediates that can be activated by Lewis acids and undergo nucleophilic substitution, providing a versatile strategy to functionalize the α-position of amines.^22^ Although the mechanism of the Shono oxidation has been investigated through cyclic voltammetry and computational studies, to the best of our knowledge, analysis by *operando* NMR spectroscopy has not yet been reported.^23^ Thus, the Shono oxidation was selected as an ideal benchmark reaction to evaluate the capabilities of our *operando* EC-NMR system to probing mechanistic pathways in electrochemical transformations.

With the Shono oxidation selected as a representative system, we performed an electrochemical oxidation of *N-*tosyl (Ts)-pyrrolidine **4** under *operando* EC-NMR conditions. In this transformation, anodic oxidation generates the reactive iminium intermediate **INT3**, which is subsequently trapped by MeOH to form the *N,O*-acetal product **5** (Figure 3A).^24^ To assess the feasibility of using less expensive, nondeuterated solvents for the *operando* analysis, the reaction was carried out in nondeuterated MeCN with solvent suppression, acquiring a single-scan ^1^H NMR spectrum every minute over the course of 2.5 hours (Figure 3B).

**Figure 3:**
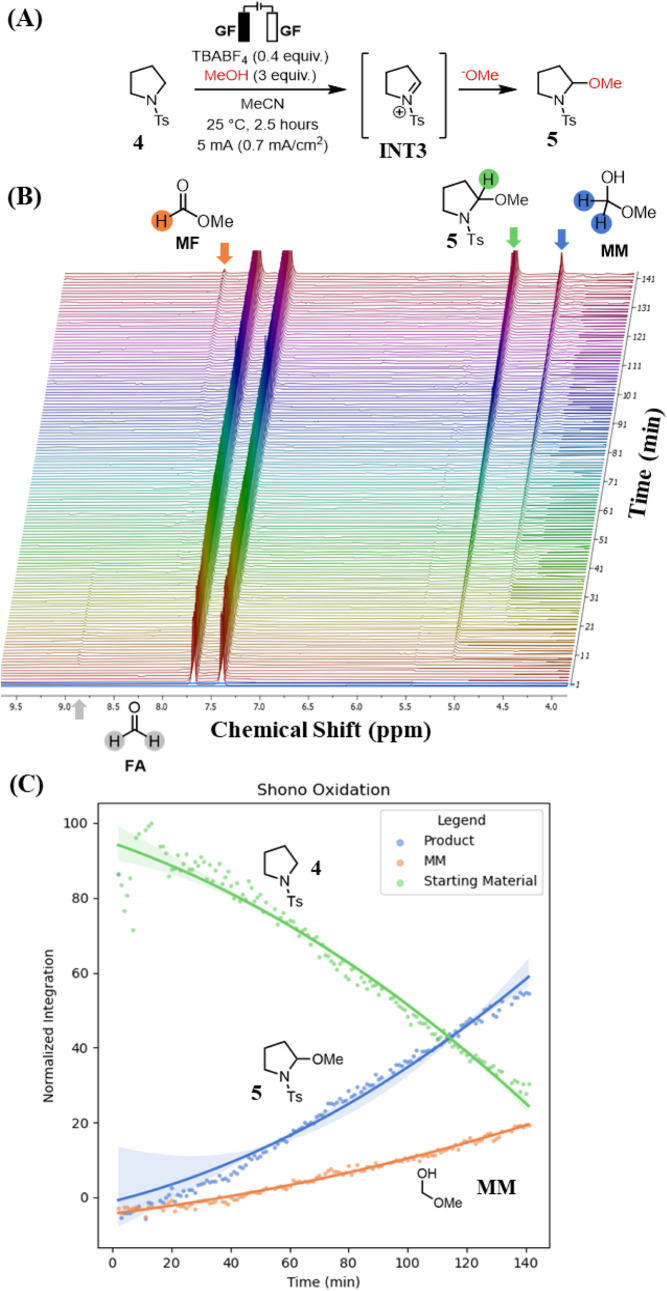
(A) Shono oxidation of Ts-pyrrolidine **4**. (B) Stacked spectra of the Shono oxidation of **4**. (C) Normalized integral graph of stacked spectra. Error bars (95% CI) are wide, suggesting lower precision of the mean estimate at the start of the experiment.

Product **5** was first detected at ∼9 min, with its 5.09 ppm signal steadily increasing in intensity throughout the course of the reaction (Figure 3B). Interestingly, a new peak at 4.53 ppm appeared approximately 20 min after the start of the data collection but did not persist in the postreaction NMR spectrum of the crude sample after solvent removal, suggesting the formation of a transient species. Based on these findings, we hypothesized that MeOH undergoes anodic oxidation to formaldehyde (FA) that is observable at 8.72 ppm, which then reacts with another equivalent of MeOH to yield methoxymethanol (MM) as the identity of the peak at 4.53 ppm. After ∼100 min, a new signal emerged at 8.09 ppm, consistent with further oxidation of MM to methyl formate (MF), as supported by literature ^1^H NMR data for both MM and MF.^25^ Taken together, these observations reveal an unproductive pathway in which MeOH is consumed via anodic oxidation. This case study underscores the value of real-time monitoring of electrochemical reactions to identify off-cycle processes and inform rational optimization.

### Electrochemical Reductive Coupling

Reductive cross-coupling reactions are widely used in both academic and industrial setting for the rapid and efficient formation of C–C and C–X bonds.^26^ Recent advances have enabled highly selective reductive couplings of electrophiles using cobalt and nickel catalysts in combination with metal reductants such as manganese and zinc, eliminating the need for sensitive organometallic reagents.^27^ Photochemical strategies have further expanded the scope of reductive coupling, allowing diverse C–C bond-forming processes when used in conjunction with Ni catalysis.^28^ However, these approaches rely on specific metal reductants or photosensitizers with fixed reduction potentials.^29^ Electroreductive coupling offers a practical and versatile alternative, providing precise control over a broad range of reduction potentials.^30^ To provide a way to deepen the mechanistic understanding of these reactions, we studied an electroreductive coupling reaction using *operando* EC-NMR.

In 2022, Zeng and coworkers reported an electrochemical radical–radical cross-coupling that converts unactivated imines and alkyl nitriles into β-amino nitriles (Figure 4A).^31^ Using readily available imines and MeCN as the nitrile source, they synthesized a broad range of β-amino nitriles, which are motifs frequently found in natural products and bioactive compounds.^32^ We adapted this transformation to our recirculating flow electrochemistry setup, coupling imine **6** with MeCN to form β-amino nitrile **7**, while monitoring the reaction in real time using operando EC-NMR. The experiment was performed in nondeuterated MeCN with solvent suppression, collecting an ^1^H NMR spectrum every 3 min over 7 hours (Figure 4B).

**Figure 4:**
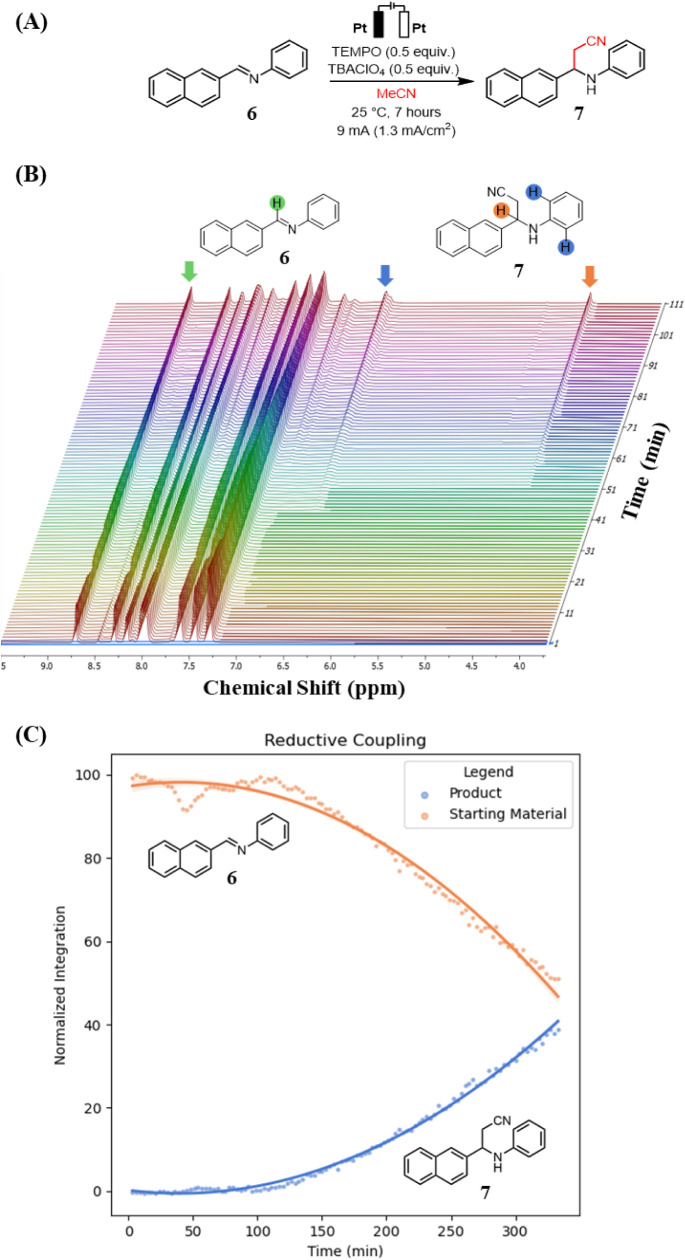
(A) Electrochemical reductive coupling of imine **6** with MeCN. (B) Stacked spectra of electrochemical reductive coupling of imine **6** with MeCN. (C) Normalized integral graph of stacked spectra.

An induction period of ∼100 min was observed before the formation of any detectable product. After this point, the signals corresponding to the product **7** at 4.51 and 6.68 ppm gradually increased in magnitude over the next 230 min. This behavior suggests that under the conditions of constant current, the system must first reach the reduction potential of the imine before the onset of the reductive coupling reaction process. These findings suggest that increasing the temperature or the applied current would be reasonable strategies to accelerate the reaction rate. More broadly, this study highlights the robustness of *operando* EC-NMR technique, which provided stable, high-quality and reliable data acquisition over an extended monitoring time period.

## Conclusions

The *operando* EC-NMR system adapted from the InsightMR® flow unit provides an accessible, straightforward, and nonintrusive approach for real-time analysis of electrochemical reactions. This method was used to elucidate the chemoselectivity in electrooxidative allene dioxygenations, as well as deliver valuable mechanistic insights into the Shono oxidation and reductive coupling reactions. Moving forward, this *operando* EC-NMR is expected to be a convenient and powerful enabling technology for mechanistic discovery and rational optimization in the development of electrochemical reactions.

## Experimental Section

### General Information

Electrochemical reactions were performed in Schlenk tubes. Unless otherwise specified, reagents were used as obtained from Sigma-Aldrich, Oakwood Products, Alfa Aesar, Combi-Blocks, Acros Organics, or Chem-Impex and used directly without further purification. 2,2,6,6-Tetramethyl-1-oxo-piperidinium tetrafluoroborate (TEMPO+), 1-methyl-1-phenylallene, 1-tosylpyrrolidine, and (*E*)-1-(naphthalen-2-yl)-*N*-phenylmethanimine and their respective products were synthesized according to the literature.^15,24b,31a,33^ Platinum wire (diameter: 0.254 mm × length: 14 mm, +99.99%, obtained from Strem) and graphite felt (length: 20 mm × width: 10 mm × height: 5 mm, Fuel Cell Earth) were connected using a house-made stainless steel electrode holder. Electrolysis was conducted using a house-made power supply in constant current mode. For detailed information, see the Supporting Information. Acetonitrile was dispensed from an Inert PureSolv PS-MD-5 solvent purification system. In all cases where the use of TEMPO+ is mentioned, the salt TEMPO^+^BF_4_^–^ form was employed. ^1^H NMR spectra were obtained using the Bruker Avance Neo 500 spectrometer.^17^

### Electrooxidative Dioxygenation

A Schlenk tube under N_2_ was charged with potassium hexafluorophosphate (110 mg, 0.6 mmol, 3.0 equiv), water (0.4 mL), *d*-MeCN (8 mL), TEMPO (63 mg, 0.4 mmol, 2 equiv), and allene **1** (0.03 mL. 0.2 mmol). The tube was capped with a rubber septum with graphite felt (GF) attached to the anode and Pt wire attached to the cathode (see Figure S1). A balloon of N_2_ was attached to the Schlenk tube. The reaction was set up as shown in Figure S8 and the temperature of the thermostated jacket was set to 24 °C. The HPLC pump lines were inserted into the rubber septum where the inlet line was submerged in the solution and the outlet line hovered over the solution. The pump was set to circulate the solution at a rate of 2 mL/min through the transfer line to the probe and back to the reaction mixture. Upon confirmation of the presence of **1** in the probe via ^1^H NMR, a constant current of 12 mA was applied to the system over 1 h. At the same time, one scan standard ^1^H NMR spectra was taken every minute using Program A (see SI) to measure the formation of allylic alcohol **2** and diene **3** over time. Upon completion, the pump lines were taken out of the reaction vessel, and the setup was washed with MeCN at 8 mL/min for 10 min. The electrodes were washed with acetone and the volatiles concentrated via rotary evaporation. The data was processed in the MestReNova software.

### Shono Oxidation

A Schlenk tube under N_2_ was charged with tetrabutylammonium tetrafluoroborate (132 mg, 0.4 mmol, 0.4 equiv), MeCN (8 mL), MeOH (0.12 mL, 3.0 mmol, 3 equiv), and 1-tosylpyrrolidine **4** (225 mg, 1.0 mmol). The tube was capped with a rubber septum with two GF electrodes and a balloon of N_2_ was attached to the Schlenk tube. The reaction was set up and the temperature of the thermostated jacket was set to 24 °C. The HPLC pump lines were inserted into the rubber septum where the inlet line was submerged in the solution and the outlet line hovered over the solution. The pump was set to circulate the solution at a rate of 2 mL/min through the transfer line to the probe and back to the reaction mixture. Upon confirmation of the presence of **4** in the probe via ^1^H NMR, a constant current of 5 mA was applied to the system over 2.5 h. At the same time, a ^1^H NMR was taken every minute with 1 scan using Program B to measure hemiaminal **5** formation over time. Upon completion, the pump lines were taken out of the reaction vessel, and the probe was washed with MeCN at 8 mL/min for 10 min. The electrodes were washed with acetone and the volatiles concentrated via rotary evaporation. The data was processed in the MestReNova software.

### Reductive Imine Coupling

A Schlenk tube under N_2_ was charged with tetrabutylammonium perchlorate (144 mg, 0.42 mmol, 0.5 equiv), MeCN (8 mL), TEMPO (66 mg, 0.42 mmol, 0.5 equiv), and imine **6** (194 mg, 0.84 mmol). The tube was capped with a rubber septum with two Pt wire electrodes and a balloon of N_2_ was attached to the Schlenk tube. The reaction was set up and the temperature of the tube was set to 24 °C. The HPLC pump lines were inserted into the rubber septum where the inlet line was submerged in the solution and the outlet line hovered over the solution. The pump was set to circulate the solution at a rate of 2 mL/min through the transfer line to the probe and back to the reaction mixture. Upon confirmation of the presence of starting material in the probe via ^1^H NMR, a constant current of 9 mA was applied to the system over 7 h. At the same time, a ^1^H NMR was taken every 3 min with 1 scan using Program B to measure the formation of β-aminonitrile **3.7** over time. Upon completion, the pump lines were taken out of the reaction vessel, and the probe was washed with MeCN at 8 mL/min for 10 min. The electrodes were washed with acetone and the volatiles concentrated via rotary evaporation. The data was processed in the MestReNova software.

## Supplementary Material



## Notes

Reaction setup; collecting and analyzing data; full ^1^H NMR spectra for all experiments (PDF)

## §

Farmabios S.p.a., I-27027, Gropello Cairoli (PV), Italy

## ∥

Department of Chemistry, Cornell University, Ithaca, New York, 14853, United States.
