# Supplementary material for: Operando NMR Monitoring of Electrochemical Reactions
Source: Org Process Res Dev. 2026 Apr 23;30(5):1189–96. doi: 10.1021/acs.oprd.5c00483 (PMC13185111; doi:10.1021/acs.oprd.5c00483)
Supplement: Supplementary file 1 [file op5c00483_si_001.pdf]

Supporting Information

Operando NMR Monitoring of Electrochemical Reactions

Ken S. Lee,[1] Federico Barbieri,[2,†] Colin S. Crawford,[1,††] Heike Hofstetter,[1] Jennifer M. Schomaker[1,*]

[1] Department of Chemistry, University of Wisconsin, Madison, Wisconsin 53706, United States.
[2] Department of Chemistry, University of Pavia, Pavia, Lombardy 12-27100, Italy

*Corresponding author. Email: schomakerj@chem.wisc.edu

†Current address: Farmabios S.p.a., I-27027, Gropello Cairoli (PV), Italy.

††Current address: Department of Chemistry, Cornell University, Ithaca, New York, 14853, United States.

Table of contents

I. General information.. S2
II. Reaction setup………………………………... S3
III. *Operando* EC NMR setup.. S5
IV. Experimental procedures.. S8
V. Full spectra.. S11
VI. Experimental references... S13

I. General information

Electrochemical reactions were performed in Schlenk tubes. Unless otherwise specified, reagents were used as obtained from Sigma-Aldrich, Oakwood Products, Alfa Aesar, Combi-Blocks, Acros Organics, or Chem-Impex and used directly without further purification. 2,2,6,6-Tetramethyl-1-oxo-piperidinium tetrafluoroborate (TEMPO+), 1-methyl-1-phenylallene, 1-tosylpyrrolidine, and (E)-1-(naphthalen-2-yl)-N-phenylmethanimine and their respective products were synthesized according to the literature.[1-4] Platinum wire (diameter: 0.254 mm × length: 14 mm, 99.99+%, obtained from Strem) and graphite felt (length: 20 mm × width: 10 mm × height: 5 mm, Fuel Cell Earth) were connected using a house-made stainless steel electrode holder. Electrolysis was conducted using a house-made power supply in constant current mode. For detailed information, see the discussion below. Acetonitrile was dispensed from an Inert PureSolv PS-MD-5 solvent purification system. In all cases where the use of TEMPO+ is mentioned, the $TEMPO^{+}BF_4^{-}$ form of the salt was employed. ^{1}H NMR spectra were obtained using the Bruker Avance Neo 500 spectrometer.[5] The NMR facilities are funded by the NSF (CHE-1048642 and CHE-2017891). The purchase of the Thermo Q Exactive™ Plus in 2015 was funded by NIH Award 1S10 OD020022-1 to the Department of Chemistry.

II. Reaction setup

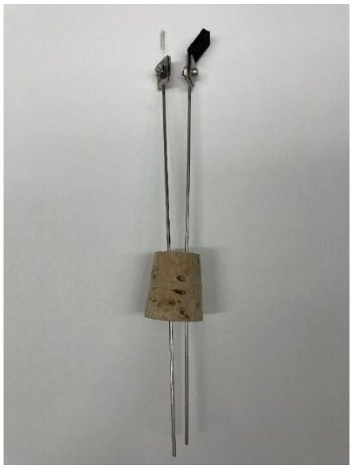

Figure S1. Steve Myers of the Machine Shop in the Department of Chemistry at UW-Madison manufactured the electrode holders (15.0 mm x 10.0 mm x 1.0 mm), modeled after Ackermann's protocol report.[6] A graphite felt and a platinum plate should be tightly held by the stainless electrode holder. Septum used instead of cork in *operando* EC NMR experiments.

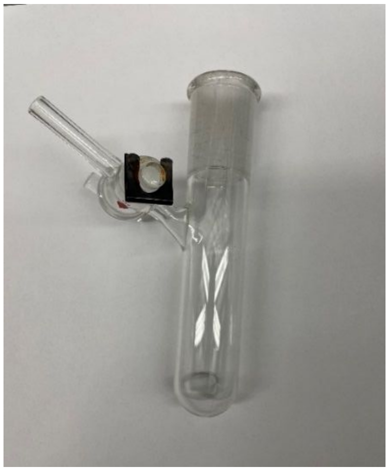

Figure S2. Tracy Drier in the glass shop in the Department of Chemistry at UW–Madison manufactured the Schlenk tubes.

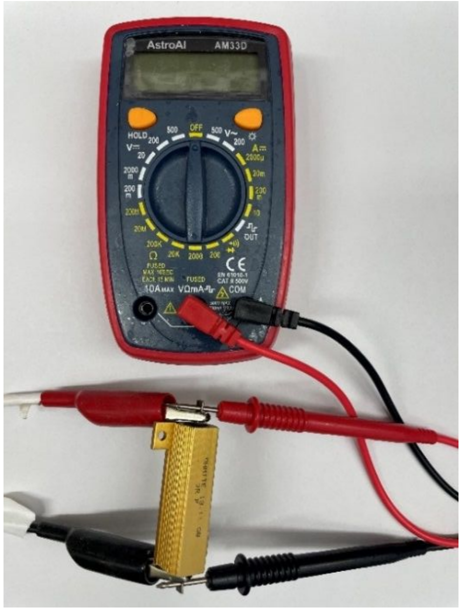

Figure S3. The current was checked using an electrical multimeter with a resistor (10 Ω).

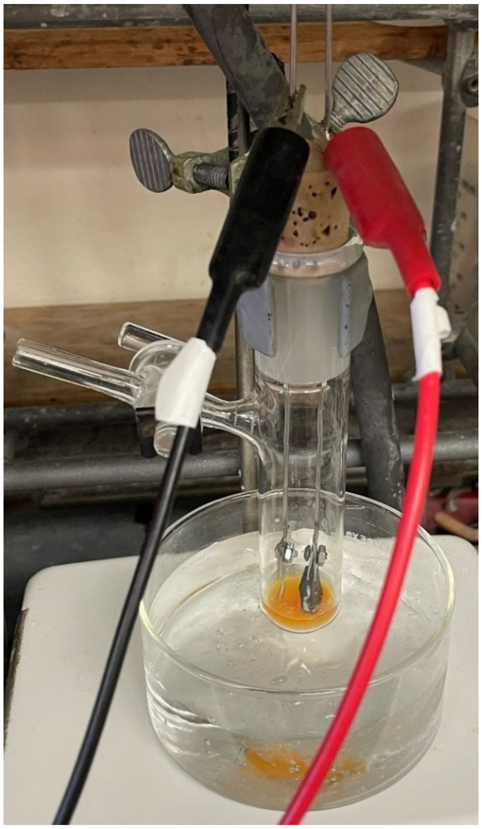

Figure S4. A typical reaction setup of batch experiments.

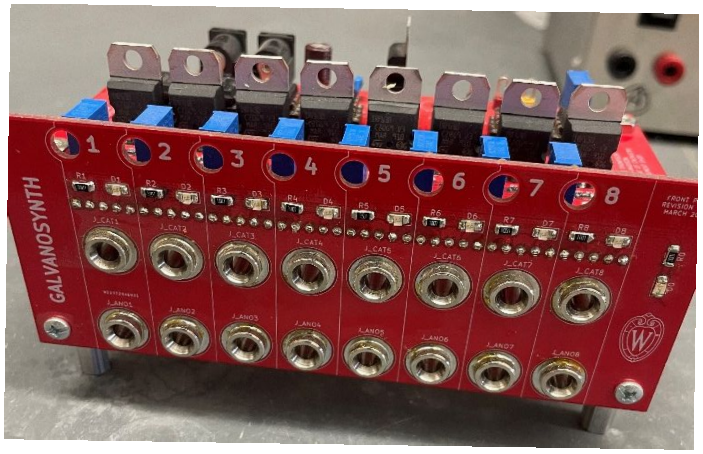

Figure S5. Power supply: original design and fabrication by Dr. Blaise J. Thompson.[7]

III. *Operando* EC NMR setup

Insight MR[8]: After disabling the autosampler, the InsightMR flow tube assembly (4 meters) is inserted into the spectrometer. An Azura P4.1 pump (Knauer, Berlin, Germany) with a stainless-steel pump head containing Hastelloy-C inlays for corrosive media is used to circulate the solution from the reaction vessel through the transfer line and back at 2 mL/min (for better shimming). The transfer line in a thermostated jacket and the probe were kept at the same temperature. For temperature control of the line a Julabo F25 refrigerated and heated circulator (Julabo, Seelbach, Germany) containing a water/glycol mixture. Polytetrafluoroethylene (PTFE) teflon HPLC tubing (O.D. x I.D. x L: 1.6 mm x 1 mm x 1.4 m) was used to transfer the solution to the pump. Total distance from the reaction vessel to the NMR is 5.4 m (4 m flow tube + 1.4 m PTFE tubing) where solvent usage scales with distance.

NMR data acquisition: All experiments were carried out on a Neo-500 MHz NMR spectrometer (Bruker Biospin Corp., Billerica, MA, USA) equipped with a 5mm Prodigy LN2 cryoprobe with a z-gradient using Insight2.0 software in Topspin.4.3 (11.8ms ^{1}H p1).

Deuterated solvents (Program A): ^{1}H NMR experiments were carried out in deuterated solvents using a standard zg30 pulse program. The experiments were set up under automation in Bruker's insight NMR software. The experiments were run locked with ns = 1 and aq = 3.7s. All NMR data were collected with a 14.7 kHz sweep width, the transmitter frequency was centered at 6.25ppm on the ^{1}H channel. Experiments were set up using Insight2.0, a package based on the automation software IconNMR. Data was processed in MestReNova (version 15.0.0, Mestrelab Research, Santiago de Compostela, Spain) and visualized using a stack plot and analyzed with integral plots. The direct dimension was 8192 complex points that were zero filled 4 times. An exponential window function with 0.3 Hz line broadening was applied to all 1D time-domain data.

Non-deuterated solvents (Program B): ^{1}H NMR experiments were carried out in non-deuterated solvents using a 1d noesy pulse sequence with a shaped pulse for presaturation and cw decoupling on f2 (Bruker pulse program lc1pncwgpps). The experiments were set up under automation based on the parameter set LC1PNCWGPPS. Automation will acquire 2 spectra, a preparation experiment that is used to determine parameters such as O1, to automatically shim on a solvent, and to create the selective pulse, and then an optimized ^{1}H spectrum with solvent suppression. The delay d1 was set to 3s, the acquisition time 0.56s.

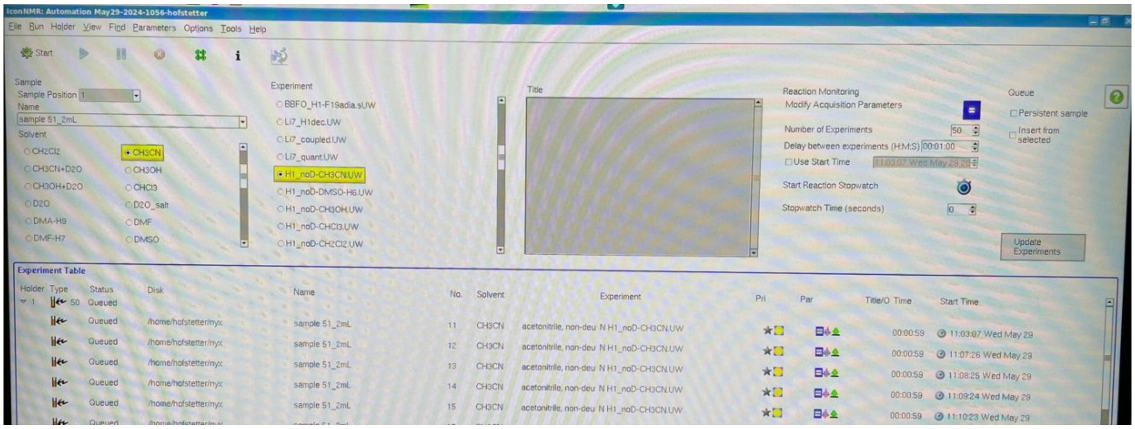

Figure S6. Insight 2.0 software IconNMR example experiment.

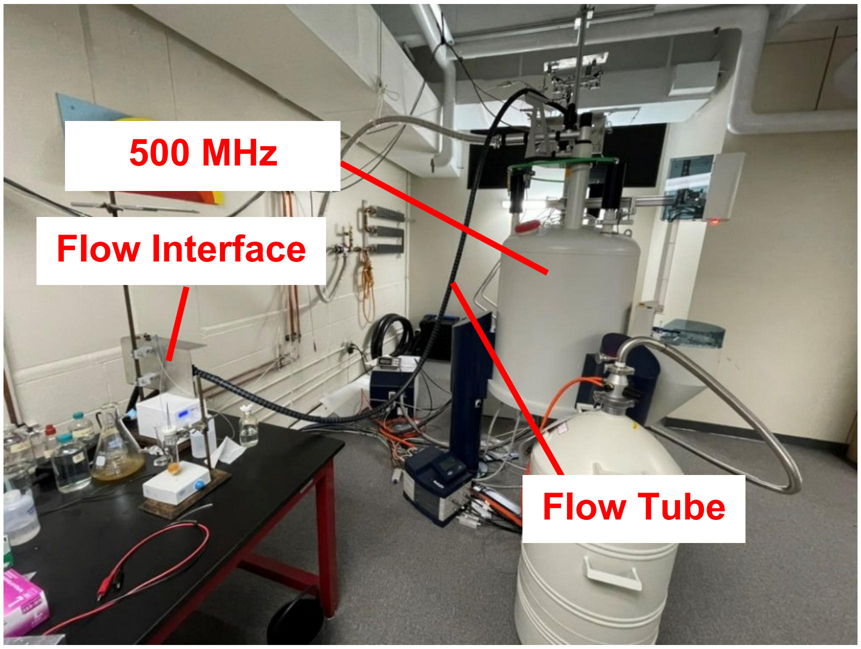

Figure S7. Overall setup.

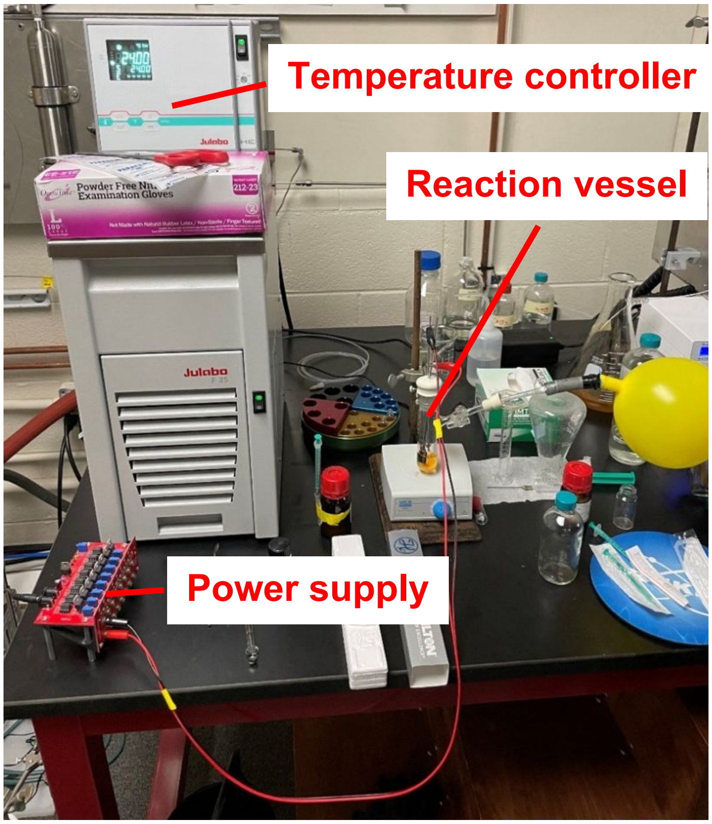

Figure S8. EC NMR Reaction setup.

IV. Experimental procedures.

Allene Dioxygenation

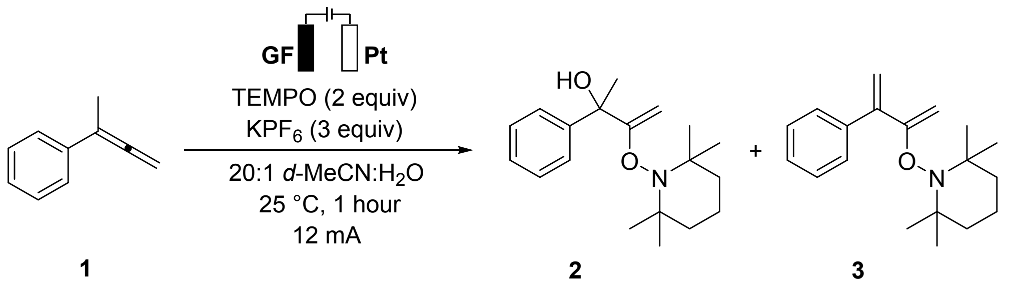

To a Schlenk tube under N_2 conditions was added potassium hexafluorophosphate (110 mg, 0.6 mmol, 3.0 equiv), water (0.4 mL), d-MeCN (8 mL), TEMPO (63 mg, 0.4 mmol, 2 equiv), and allene **1** (0.03 mL. 0.2 mmol). The tube was capped with a rubber septum with graphite felt (GF) attached to the anode and Pt wire attached to the cathode (see **Figure S1**). A balloon of N_2 was attached to the Schlenk tube. The reaction was set up as shown in **Figure S8** and the temperature of the tube was set to 24 °C. The HPLC pump lines were inserted into the rubber septum where the inlet line was submerged in the solution and the outlet line hovered over the solution. The pump was set to circulate the solution at a rate of 2 mL/min through the transfer line to the probe and back to the reaction mixture. Upon confirmation of the presence of starting material in the probe via ^{1}H NMR, a constant current of 12 mA was applied to the system over 1 hour. At the same time, a standard ^{1}H NMR was taken every minute with 1 scan using **Program A** to measure the formation of allylic alcohol **2** and diene **3** over time. Upon completion, the pump lines were taken out of the reaction vessel, and the probe was washed with MeCN at 8 mL/min for 10 minutes. The electrodes were washed with acetone and the volatiles concentrated via rotary evaporation. The data was processed in the MestReNova software.

Shono oxidation

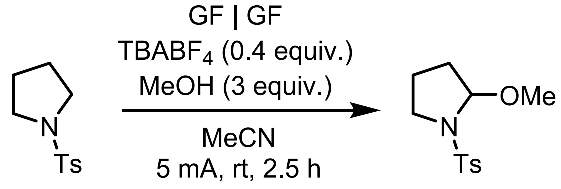

4 5

To a Schlenk tube under N_2 conditions was added tetrabutylammonium tetrafluoroborate (132 mg, 0.4 mmol, 0.4 equiv), MeCN (8 mL), MeOH (0.12 mL, 3.0 mmol, 3 equiv), and 1-tosylpyrrolidine **4** (225 mg, 1.0 mmol). The tube was capped with a rubber septum with two GF electrodes (see **Figure S1**) and a balloon of N_2 was attached to the Schlenk tube. The reaction was set up as shown in **Figure S8** and the temperature of the tube was set to 24 °C. The HPLC pump lines were inserted into the rubber septum where the inlet line was submerged in the solution and the outlet line hovered over the solution. The pump was set to circulate the solution at a rate of 2 mL/min through the transfer line to the probe and back to the reaction mixture. Upon confirmation of the presence of starting material in the probe via ^{1}H NMR, a constant current of 5 mA was applied to the system over 2.5 hours. At the same time, a ^{1}H NMR was taken every minute with 1 scan using **Program B** to measure the formation of hemiaminal **5** over time. Upon completion, the pump lines were taken out of the reaction vessel, and the probe was washed with MeCN at 8 mL/min for 10 minutes. The electrodes were washed with acetone and the volatiles concentrated via rotary evaporation. The data was processed in the MestReNova software.

Reductive coupling

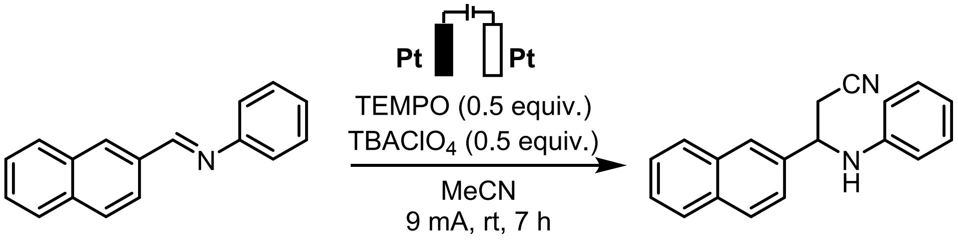

6 **7**

To a Schlenk tube under N_2 conditions was added tetrabutylammonium perchlorate (144 mg, 0.42 mmol, 0.5 equiv), MeCN (8 mL), TEMPO (66 mg, 0.42 mmol, 0.5 equiv), and imine **6** (194 mg, 0.84 mmol). The tube was capped with a rubber septum with two Pt wire electrodes (see **Figure S1**) and a balloon of N_2 was attached to the Schlenk tube. The reaction was set up as shown in **Figure S8** and the temperature of the tube was set to 24 °C. The HPLC pump lines were inserted into the rubber septum where the inlet line was submerged in the solution and the outlet line hovered over the solution. The pump was set to circulate the solution at a rate of 2 mL/min through the transfer line to the probe and back to the reaction mixture. Upon confirmation of the presence of starting material in the probe via ^{1}H NMR, a constant current of 9 mA was applied to the system over 7 hours. At the same time, a ^{1}H NMR was taken every 3 minutes with 1 scan using **Program B** to measure the formation of β-aminonitrile **7** over time. Upon completion, the pump lines were taken out of the reaction vessel, and the probe was washed with MeCN at 8 mL/min for 10 minutes. The electrodes were washed with acetone and the volatiles concentrated via rotary evaporation. The data was processed in the MestReNova software.

V. Full spectra

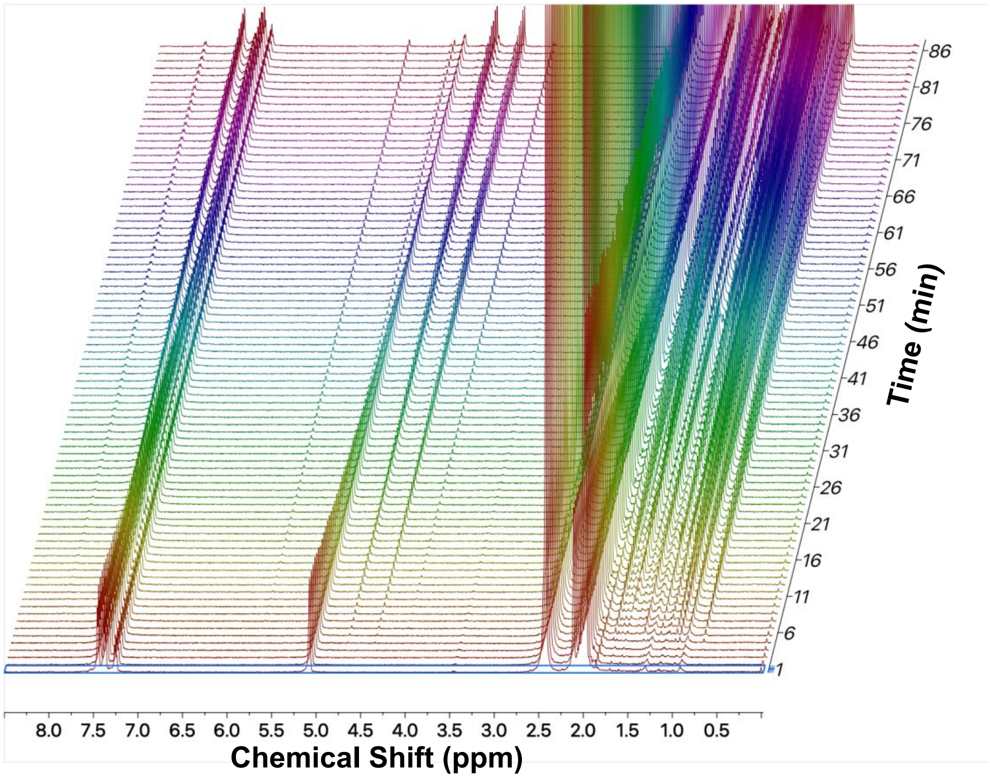

Figure S9. Allene dioxygenation *operando* EC NMR full spectra: 20:1 CD_3CN:H_2O

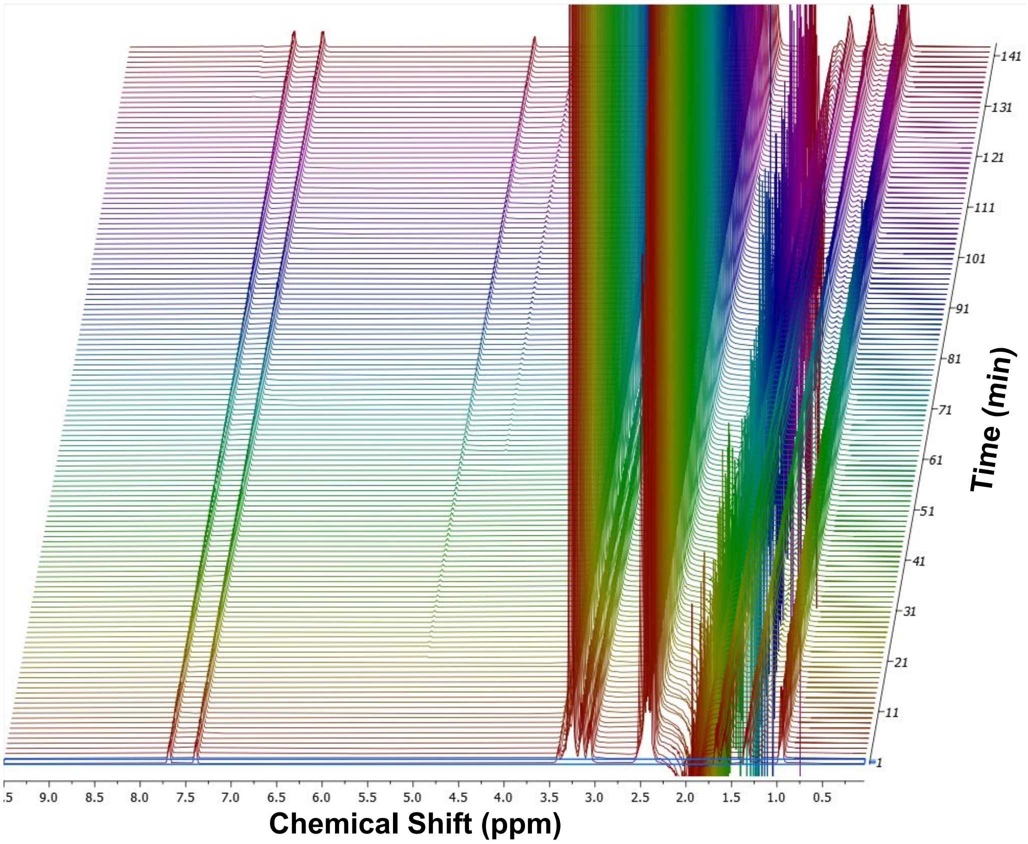

Figure S10. Shono oxidation *operando* EC NMR full spectra: MeCN solvent suppression

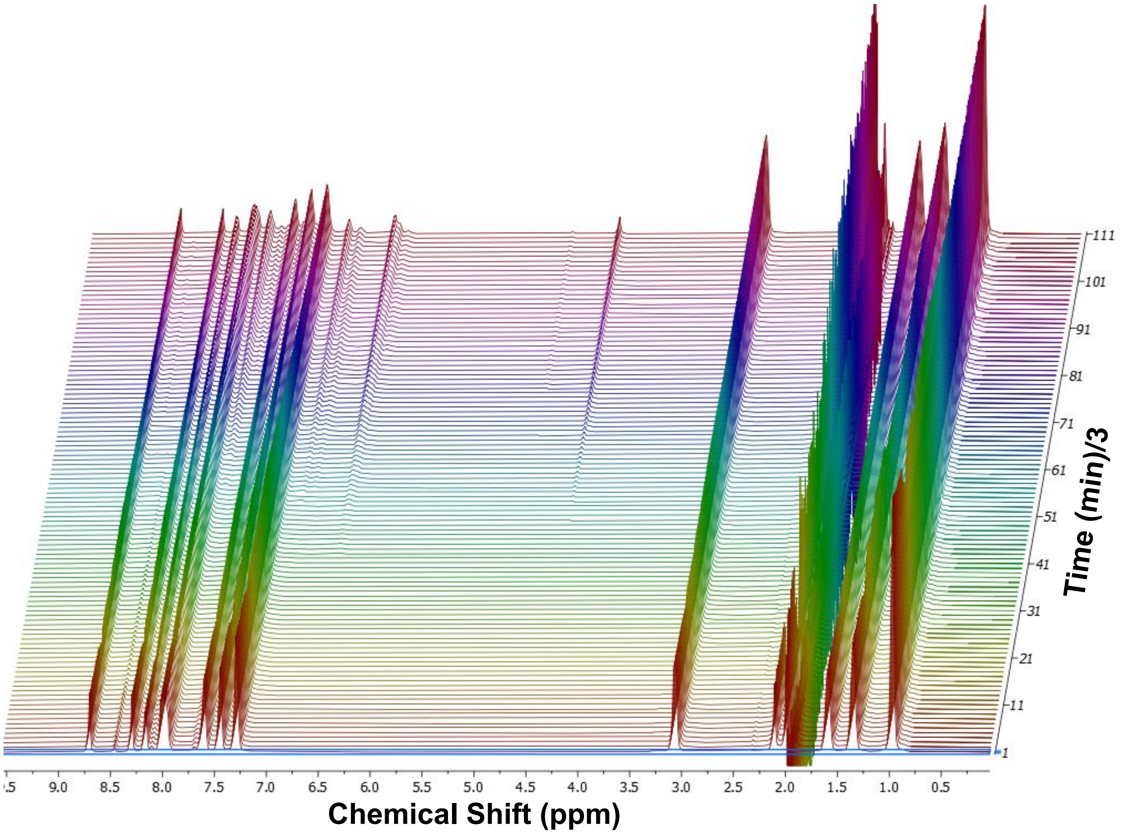

Figure S11. Reductive coupling *operando* EC NMR full spectra: MeCN solvent suppression

VI. Experimental references

(1) Wang, J.; Yang, S. A silver triflate-catalyzed cascade of in situ-oxidation and allylation of arylbenzylamines. *Tetrahedron Lett.* **2016**, *57*, 3444–3448.

(2) Lee, K. S.; Barbieri, F.; Casali, E.; Marris, E. T.; Zanoni, G.; Schomaker, J. M. Elucidating the Mechanism of Electrooxidative Allene Dioxygenation: Dual Role of Tetramethylpiperidine N-Oxyl (TEMPO). *J. Am. Chem. Soc.* **2025**, *147*, 318–330.

(3) Novaes, L. F. T.; Ho, J. S. K.; Mao, K.; Liu, K.; Tanwar, M.; Neurock, M.; Villemure, E.; Terrett, J. A.; Lin, S. Exploring Electrochemical C(sp^3)-H Oxidation for the Late-Stage Methylation of Complex Molecules. *J. Am. Chem. Soc.* **2022**, *144*, 1187−1197.

(4) Zeng, W. M.; Wang, Z. L.; He, Y. H.; Guan, Z. Electrochemical Radical-Radical Cross-Coupling: Direct Access to β-Amino Nitriles from Unactivated Imines and Alkyl Nitriles. *Green Chem.* **2022**, *24*, 4928–4934.

(5) UW-Madison NMR page. https://nmr.chem.wisc.edu/nmr-spectrometers/. (accessed October 27, 2025)

(6) Tian, C.; Meyer, T. H.; Stangier, M.; Dhawa, U.; Rauch, K.; Finger, L. H.; Ackermann, L. Cobaltaelectro-catalyzed C–H activation for resource-economical molecular syntheses *Nature Protocols* **2020**, *15*, 1760-1774.

(7) Cowper, N.; Chernowsky, C.; Williams, O.; Wickens, Z. Potent Reductants via ElectronPrimed Photoredox Catalysis: Unlocking Aryl Chlorides for Radical Coupling. *J. Am. Chem. Soc.* **2020**, *142*, 2093-2099.
(8) Bruker InsightMR Page. https://www.bruker.com/en/products-and-solutions/mr/nmr-software/insight-mr.html. (accessed October 27, 2025)
